# Detection of Histamine Based on Gold Nanoparticles with Dual Sensor System of Colorimetric and Fluorescence

**DOI:** 10.3390/foods9030316

**Published:** 2020-03-09

**Authors:** Jingran Bi, Chuan Tian, Gong-Liang Zhang, Hongshun Hao, Hong-Man Hou

**Affiliations:** 1 School of Food Science and Technology, Dalian Polytechnic University, No. 1, Qinggongyuan, Ganjingzi District, Dalian 116034, Liaoning, China; 18900986803@163.com (C.T.); zgl_mp@163.com (G.-L.Z.); beike1952@163.com (H.H.); 2 Liaoning Key Lab for Aquatic Processing Quality and Safety, No. 1, Qinggongyuan, Ganjingzi District, Dalian 116034, Liaoning, China

**Keywords:** gold nanoparticles, histamine, UV-visible and fluorescence, visual detection, spoilage marker

## Abstract

Gold nanoparticles (Au-NPs), with the dual sensor system of colorimetric and fluorescence responses, were developed for the determination of histamine as a spoilage monitor for distinguishing lifetime and freshness of aquatic products. Upon addition of histamine, the absorption coefficient orders of magnitude via the interaction of free electrons and photons were affected, and the characteristic absorption peak of Au-NPs was red-shifted from 520 nm to 664 nm. Meanwhile, the large amino groups in the networks of histamine-Au-NPs with high molecular orbital exhibited excellent fluorescence behavior at 415 nm. Au-NPs offered a range of 0.001–10.0 μM and 0.01–1.0 μM with a limit of detection of 0.87 nM and 2.04 nM by UV-vis and fluorescence spectrum assay, respectively. Moreover, Au-NPs could be used to semiquantitatively analyze histamine with the naked eye, since the significant colorimetric and fluorescence reaction of Au-NPs solution that coincided with different concentrations of histamine can be observed as the histamine concentration was 0.1–1.0 μM. Both of the dual-sensor systems of Au-NPs were successfully applied to the quantitative analysis of histamine in fresh salmon muscle, suggesting the simplicity and rapidity in the dual detection approaches of Au-NPs might be suitable for spoilage assay of aquatic food to ensure food safety.

## 1. Introduction

Biogenic amines are a type of significant toxic substances directly involved in food quality and safety. Under improper storage conditions, it is easy to cause spoilage bacteria to grow and metabolize decarboxylase, resulting in decarboxylation of amino acids in high-protein foods (such as dairy products, seafood, meat products, etc.) to form various biological amines, mainly including histamine, tyramine, cadaverine, and putrescine [1]. In particular, histamine (4-(2-aminoethyl)-1H-imidazole) is one of the most important biogenic amines, commonly formed by free L-histidine under the action of exogenous decarboxylase produced by microbial metabolism [2]. Once formed, histamine is hard to degrade by conventional methods such as heating and freezing due to its excellent stability. Moreover, food-borne histamine as an alkaline nitrogen-containing hazardous substance can affect the respiratory system and digestive system and frequently causes food poisoning around the world [3]. Hence, many countries have established the strict guidelines of histamine to prevent histamine food poisoning. The US Food and Drug Administration (FDA) set the maximum thresholds of histamine as 500 mg/kg [4]. The European Union 2073/2005 considers the histamine limits of 100–200 mg/kg for the fishery products from fish species as safe [5]. Due to the fact that histamine level generally increases as the improper storage time extends, histamine has become a globally recognized monitor for distinguishing lifetime and freshness of aquatic products [6]. Consequently, a direct and rapid sensor for detecting histamine in food is sorely required to guarantee food safety.

For decades, various standardized laboratory analytical techniques have been applied for the determination of histamine, such as high-performance liquid chromatography (HPLC) [7], thin-layer chromatography [8], and gas chromatography–tandem mass spectrometry analysis [9]. Although these techniques are to detect histamine in biomatrix, unfortunately, they require too much laborious sample preparations, instruction expertise operations, and time-consuming procedures to be practically applied in fishery enterprises and market supervision. Hence, the establishment of a simple ultrasensitive method for histamine detection is significant and challenging.

Recently, nanosensors with the advantages of high efficiency, rapidity, and sensitivity gradually caused great concern in many areas, such as clinical diagnosis [10], pesticide residue analysis [11], and microbiological detection [12]. Due to the food industry inextricably linked to public health, more and more attention has focused on nanosensors for the determination of food freshness to guarantee public health safety [13].

Although histamine does not absorb strongly, it can be detected by the UV-vis spectrum after derivatization transforms or chemosensory combination. Numerous receptors have been successfully designed to detect biomolecules by hydrogen bonds [14,15]. However, water molecules typically compete with analytes for binding to receptors in aqueous solutions [16]. Hence, metal ions are regarded as an appropriate probe for forming complexes with biomolecules by electrostatic interaction and are suitable for bioanalysis of samples with high moisture content.

Gold nanoparticles (Au-NPs) have unique dispersibility, long-term stability, sensitive optical properties, and facile surface functionalization, and have been widely applied in biosensing determination, such as the detection of toxin [17,18], DNA [19], and amino acid [20]. However, up to now, Au-NPs probes for detecting molecular species have been based almost exclusively on colorimetric properties, lack multisensor properties, and cannot be flexibly applied in practical analysis.

In this work, by the precursor ratio, an economical and practical new Au-NPs biomarker was fabricated for the detection of histamine with dual-sensor analysis. Due to the electrostatic interaction between histamine and Au-NPs, which increases the electron density and surface amino group content, histamine can be detected by Au-NPs via the dual approaches of colorimetric and fluoroscopy with high sensitivity, selectivity, and efficiency. Moreover, Au-NPs can be used to directly quantify whether the histamine content of fresh salmon muscle meets FDA standards, in the hope that this probe will be able to develop assays for the determination of freshness and spoilage of aquatic foods to ensure food safety.

## 2. Materials and Methods

### 2.1. Materials and Reagents

Chloroauric acid tetrahydrate (HAuCl_4_·4H_2_O) was purchased from Sangon Biotech (Shanghai, China). Histamine dihydrochloride, histidine, putrescine dihydrochloride, cadaverine dihydrochloride, 2-Phenylethylamine hydrochloride, and tyramine hydrochloride were obtained from Sigma (St. Louis, MO, USA) (http://www.sigmaaldrich.com). Trisodium citrate and NaCl were purchased from Tianjin Chemical Reagent Co., Ltd. (Tianjin, China). Salmon samples were purchased from a local aquatic products market. Moreover, Milli-Q-purified water (18.2 MΩcm) was used throughout the experiments.

### 2.2. Instrumentation

The morphology analysis was measured by the transmission electron microscope (JEM-2100, JEOL, Tokyo, Japan). UV-vis absorption spectra were obtained at room temperature via a UV-vis spectrophotometer (Lambda 35, PerkinElmer, Cambridge, MA, USA). Fluorescence spectra were studied by a fluorescence spectrometer (F-2700, Hitachi, Tokyo, Japan). Moreover, Fourier transforms infrared (FTIR) spectra were recorded at room temperature on the Frontier FTIR spectrometer (PerkinElmer, Norwalk, CT, USA). Average hydrodynamic diameter and polydispersity index (PDI) were measured by Dynamic Light Scattering (DLS) using the particle size analyzer (Zetasizer 3000HSA, Malvern, UK). Surface electric properties were analyzed by zeta potential analyzer (NanoZS ZEN3600, Malvern, UK).

### 2.3. Preparation of Citrate-Capped Au-NPs

Every piece of glassware was soaked in an aqua regia solution and completely washed by purified water before use. Two milliliters of 0.85% HAuCl_4_ solution was cautiously added in 48 mL boiling water, dropwise, until the final concentration was around 1.0 mM. After stirring for 5 min, Trisodium citrate was quickly dropped into the boiled HAuCl_4_ solution with different final precursor ratios of HAuCl_4_/Na_3_Ct (1:2, 1:4, 1:6, and 1:8), followed by continuous stirring and heating for 15 min. Consequently, the color of the solution gradually changed to wine red, demonstrating that Au-NPs were successfully synthesized by reducing Au (III) to Au (0) via trisodium citrate. Then, the Au-NPs solution was cooled and stored at 4 °C for reserve.

### 2.4. Detection of Histamine

Histamine was dissolved into 0.9% NaCl solution to achieve various final concentrations and added into Au-NPs solution (*v*/*v* = 1/1) with vigorous vortex-mixing for 2 min. Then, UV-vis spectra and fluorescence spectra were measured for detecting assay. The selectivity of the Au-NPs probe was also investigated by using different biological substances containing amino groups (histamine, putrescine, cadaverine, tyramine, phenylethylamine, guanine, guanosine, thymine, inosine, adenosine triphosphate (ATP), and adenosine monophosphate (AMP)).

### 2.5. Detection of Histamine In Salmon Muscle

The fresh salmon muscle was added into 0.9% NaCl solution (*w*/*v* = 1/10) and high-speed homogenate for 2 min. After being centrifuged at 4000 rpm at 4 °C for 10 min, the supernate was spiked with histamine (various final concentrations of 0.01, 0.1, 1.0 μM) and stirred thoroughly. Then, the samples were mixed with Au-NPs solution at *v*/*v* = 1/1 and measured by UV-vis spectra and fluorescence spectra to detect the concentration of histamine. Recovery was calculated from found concentration/known concentration × 100%. Precision was calculated from standard deviation/mean × 100%. Accuracy was calculated from (found concentration − known concentration)/known concentration × 100%.

### 2.6. Statistical Analysis

All measurements in the study were done in triplicate. The results reported here were the means of the three trials. The results were expressed as means ± standard deviation (SD). All the diagrams were plotted by Origin 9.2 software (Microcal, Northampton, MA, USA).

## 3. Results and Discussion

Au-NPs were successfully synthesized by being reduced and capped via citrate in boiling water (Scheme S1) with HAuCl_4_/Na_3_Ct precursor ratio of 1:6 (Figure S1). Au-NPs had extremely small particle size, good monodispersity, and strong UV-vis absorption at 520nm, indicating that colloidal gold with intense surface plasmon resonance absorption at visible wavelengths was formed, which can be further analyzed as a sensor to detect histamine.

### 3.1. Sensing Mechanism of Au-NPs Probe

**Figure 1:**
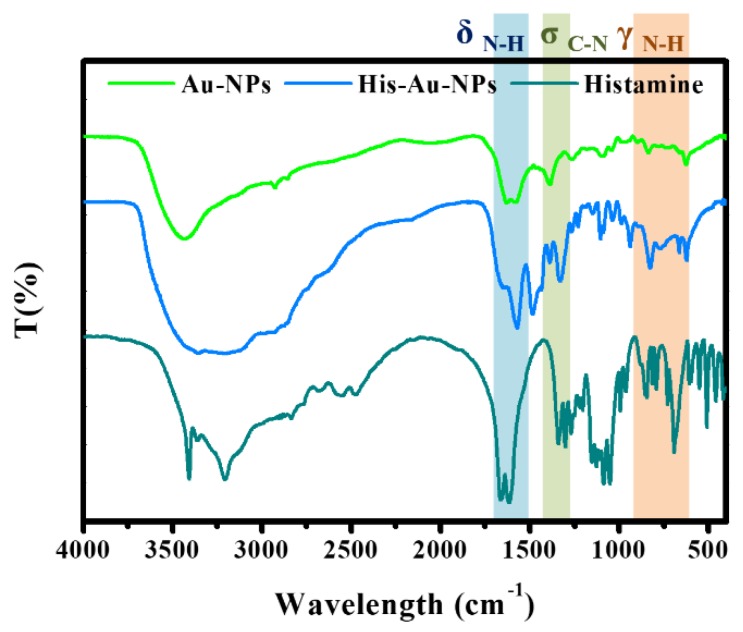
FTIR of Gold nanoparticles (Au-NPs) and the aggregation of histamine-Au-NPs.

To explore the sensing mechanism of histamine detected by Au-NPs, FTIR spectrum was applied to study the structural changes, and the results illuminated the distinct functional groups of the reaction system. As shown in Figure 1, the correlation absorption peaks of Au-NPs at 1637 cm^−1^ and 1388 cm^−1^ are attributed to σ_C=O_ and σ_C-O_, respectively, due to the decarboxylation molecules of synthetic product. Also, the strong characteristic band of histamine in the range of 1650–1600 cm^−1^ is assigned to C=N, and the absorption peak at 1564 cm^−1^ belongs to the in-plane bending vibration of the amine group. The band of 1120–1030 cm^−1^ corresponds to σ_C-N_, and the primary amine groups were observed at 850–650 cm^-1^ and attributed to γ_N-H_. After reacting with Au-NPs, the position of amine groups, including δ_C-N_, σ_C-N,_ and γ_N-H_, were remarkably strengthened; furthermore, the band of C=N double bond was also significantly enhanced, suggesting the positively charged functional groups might participate in electrostatic interactions with Au-NPs and large amine groups and unsaturated bonds on the surface of histamine-Au-NPs.

**Figure 2:**
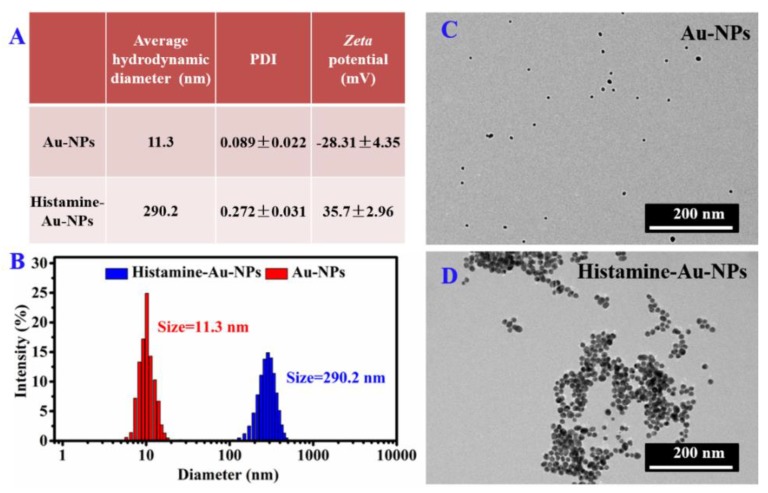
(**A**) Average hydrodynamic diameter, polydispersity index (PDI) and zeta potential information, (**B**) Dynamic Light Scattering (DLS), and (**C**–**D**) TEM images of Au-NPs and the aggregation of histamine-Au-NPs.

By DLS measure (Figure 2A,B), the average hydrodynamic diameter of Au-NPs after reaction with histamine increased from 1.3 nm to 290.2 nm, with a PDI value of 0.089 to 0.272, indicating that after the gold nanoparticles were combined with histamine, the dispersity was reduced, and the aggregation was enhanced. *Zeta* potential is consistent with DLS results, and the surface of Au-NPs is negative in charge (−28.31 ± 4.35 mV), while the histamine–Au-NPs surface is positive in charge (35.70 ± 2.96 mV) due to the NH_2_ functional group in the histamine, which enables it to attach to the negative charge of AuNPs. TEM micrograph (Figure 2C,D) also confirmed the active surface affinity of Au-NPs towards histamine. Compared with Au-NPs, the morphology of histamine-Au-NPs appears significantly aggregated.

**Scheme 1:**
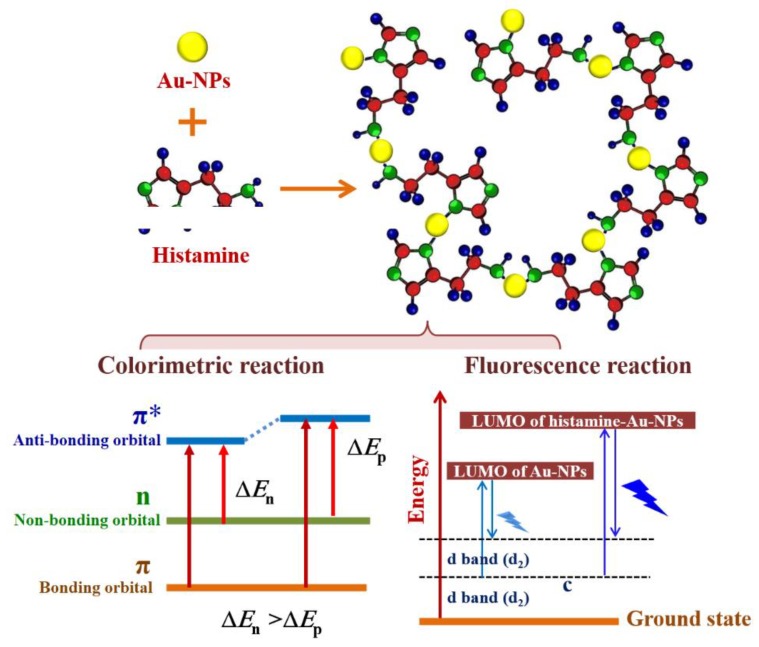
Dual sensing mechanism for determination of histamine by Au-NPs.

The key to the observed remarkable chromogenic reaction and fluorescence response is believed to be an amino role (Scheme 1). Au-NPs are negatively charged by the synthesis of Au (III) reduced and capped via citrate, and the electrostatic interaction occurred when Au-NPs are attracted by histamine that narrowed the interparticle distance between them. Histamine–Au-NPs are formed into massive networks, and the absorption coefficient orders of magnitude via the interaction of free-electrons and photons might be severely affected surface plasmon resonance absorptions enhance [21]. Moreover, the amino groups are linked with the Au-NPs, and *n*-π* conjugate occurs. Hence, the UV-vis absorption was distinctly red-shifted from wine red to dark blue, which could be observed by eye vision. Meanwhile, the large amino groups in the networks of histamine-Au-NPs have higher molecular orbital (LUMO orbital) in contrast to the original hydrogen-terminated groups of Au-NPs. As the electrons are excited, they would be relaxed to the ground state through a narrow optical band gap with the amino-fluorophore vibrations/rotations [22]. Hence, the solution of histamine-Au-NPs could exhibit excellent turn-on fluorescence behavior.

### 3.2. Analytical Performance of Au-NPs Probe

**Figure 3:**
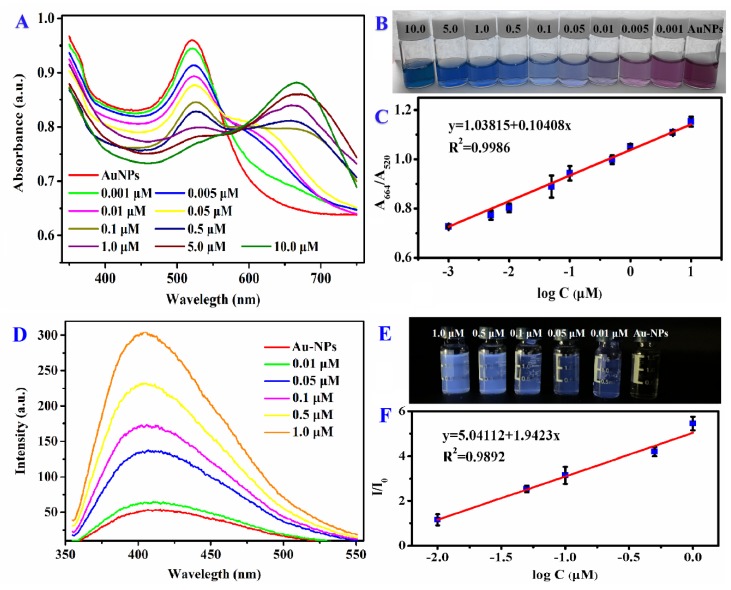
(**A**) UV–vis and (**D**) fluorescence spectra of Au-NPs with various concentrations of histamine. The corresponding (**B**) colorimetric and (**E**) fluorescent response of histamine-Au-NPs under daylight and 365nm UV-light. Linear relationships between (**C**) the absorption ratios (A_664_/A_520_), (**F**) the fluorescence intensity ratios (I/I_0_), and the logarithm value of histamine concentrations.

Compared to the wine-red Au-NPs, on the inclusion of histamine, the color of the complex solution gradually turned into dark blue, with the increased concentrations (0.001–10.0 μM) as shown in Figure 3B, that was evident enough to semiquantitatively obtain the existence of histamine by visual observation. The multicolored appearance of metallic suspension would be ascribed to their surface plasmon polaritons. The extremely tiny NPs can combine with the analyte, dramatically influencing their optical characteristics; that is to say, metallic NPs can transform the color by the change of size and dispersity. Similarly, after the gold nanoparticles reacted with histamine, the monodispersity was significantly reduced and the surface plasmon polaritons was affected.. Histamine can form a rough layer on the surface of Au-NPs through electrostatic action. The roughness of Au-NPs causes the scattering of high-momentum surface plasmons, which can be coupled with radiated light by the loss of their momentum. This process is much faster than the spontaneous recombination of exciton dipoles, so the radiation intensity enhanced [23]. In addition, the UV-vis spectrums of various concentrations of histamine detected by Au-NPs were measured, as shown in Figure 3A. The characteristic peak of Au-NPs at 520 nm was gradually weakened, and a new peak at 664 nm was progressively enhanced with the increasing concentrations of histamine from 0.001 to 10.0 μM due to the changes of electron density by complexation between histamine and Au-NPs. Furthermore, there was a tremendous linear relationship between the absorption ratios (A_664_/A_520_) and the logarithm value of histamine concentrations in the dynamic range from 0.001 to 10.0 μM with the regression equation y = 1.03815 + 0.10408x (Figure 3C). The correlation coefficient of the calibration curve was 0.9986. The detection limit (DL = 3S_b1_/S, where S_b1_ is the standard deviation of the blank solution and S is the slope of the calibration curve) was 0.87 nM.

Furthermore, by successive addition of histamine, the electrostatic interacted amino groups were progressively increased, the glaucous fluorescence of histamine-Au-NPs solution exhibited brighter and brighter under UV lamp (λ = 365 nm) (Figure 3E). As the concentration of histamine became higher than 0.1μM, the significant colorimetric and fluorescence turn-on response with Au-NPs could be captured by eye-vision. As shown in Figure 3D, histamine could also be detected by Au-NPs via fluorescence spectra analysis. The emission peak around 415 nm was strengthened gradually as the concentrations of histamine continuously increased in the range of 0.01–1.0 μM with an excellent linear relationship with the regression equation y = 5.04112x + 1.9423 (where y is the fluorescence intensity ratio between before and after adding histamine and x is the logarithm value of histamine concentrations) (Figure 3F). The correlation coefficient of the calibration curve was 0.9986. Moreover, the Au-NPs probe for detecting histamine performed a limit of detection as 2.04 nM.

Previously, HPLC was considered a typical quantitative analysis for biogenic amines, including histamine with the sensitive linear range from 2.5 to 100 ppm [24]. By modification, an HPLC method coupled with fluorescence detection was developed with linear over the range of 0.25–20 µg/mL for histamine and the detection limit of 75.0 μg/mL [7]. Kumar, N. [15] fabricated the silver nanoparticles capped with graphite as a sensor for determination of histamine and evaluated the linearity of 1–500 μM with the detection limit of 0.049 μM. Additionally, Leena Mattsson [24] applied a competitive, fluorescent, molecularly imprinted polymer (MIP) assay to detect histamine within the optimal linear range of 1–430 µM. Correspondingly, our Au-NPs probe provides a sensitive detection method of histamine via dual approaches. By the electrostatic reaction with histamine, the changes of electron density and molecular orbital induce the sensitive responses of Au-NPs, leading to the strong plasmon resonance absorption and amino-fluorophore vibrations/rotations. Moreover, Au-NPs offer a relatively broad range of 0.001–10.0 μM and 0.01–1.0 μM with a limit of detection of 0.87 nM and 2.04 nM by UV-vis and fluorescence spectrum assay, respectively. Moreover, the simplicity and rapidity of Au-NPs probes are two advantage factors in the detection process compared to the complex pretreatment process of traditional assays.

### 3.3. Selectivity of Au-NPs Probe

**Figure 4:**
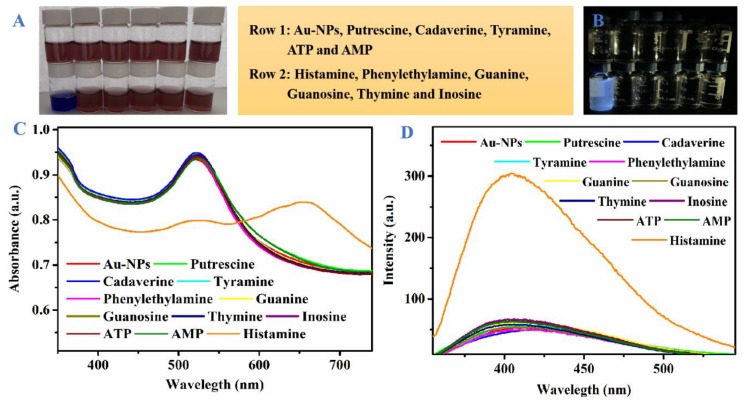
(**A**) colorimetric, (**B**) fluorescent response, (**C**) UV-vis spectra and (**D**)fluorescence spectra of Au-NPs added with different biological substances containing amino groups (histamine, putrescine, cadaverine, tyramine, phenylethylamine, guanine, guanosine, thymine, inosine, ATP, and AMP) at concentrations of 1.0 μM.

The chromogenic reaction and fluorescence response of Au-NPs was measured with various biological substances containing amino groups (histamine, putrescine, cadaverine, tyramine, phenylethylamine, guanine, guanosine, thymine, inosine, adenosine triphosphate (ATP), and adenosine monophosphate (AMP)) at concentrations of 1.0 μM each (Figure 4). Interestingly, the color of Au-NPs dramatically changed from wine red to dark blue by the target analytes of histamine, whereas other various biological substances containing amino groups still maintained their color (Figure 4A), likely due to histamine having a relatively high polar surface area, causing the bonding with Au-NPs. Meanwhile, the special UV-vis absorption spectra of Au-NPs bonded to histamine red-shifted from 520 nm to 664 nm, in sharp contrast with other biological substances, possibly due to the aggregation of Au-NPs induced by histamine (Figure 4C). By the electrostatic interactions and hydrogen-bonding between the negatively charged and positively charged histamine, the special UV-vis absorption peak of Au-NPs was red-shifted. Furthermore, only the solution of histamine-Au-NPs stood out in the experimental group with bright glaucous color under the UV-lamp (λ = 365 nm) (Figure 4B), and only the emission peak of that was significantly enhanced at 415 nm (Figure 4D), due to the fact that the electron density might have been increased by electrostatically attracted amino groups. Hence, histamine could be detected explicitly by Au-NPs via dual approaches of colorimetric and fluorometric perspectives.

### 3.4. Detection of Histamine in Salmon Muscle

For further evaluation of the effectiveness of the Au-NPs probe in the real sample, the Au-NPs probe was experimentally applied to detect three various concentrations of histamine (0.01, 0.1, and 1.0 μM) in fish muscle (Table 1). Although there is still negligible interference in the actual sample analysis, the Au-NP method does not require complicated pretreatment and therefore has excellent stability. Au-NPs probe showed a satisfactory recovery value of 96.01%–103.43% with measurement precision (RSD < 10%) and accuracy (±4%), indicating Au-NPs probe can be competent with respect to the FDA and EU requirements for histamine testing in aquatic products. In addition, the application of Au-NPs probe analysis has simple preprocessing, suitable equipment requirements, and low experimental costs; thus, the efficient and convenient Au-NPs probe has potential application value in freshness and spoilage assay of aquatic food to ensure food safety.

## 4. Conclusions

In summary, by adjusting the precursor ratio, an economical and practical Au-NP was fabricated to detect histamine via dual approaches of colorimetric and fluorescence perspectives with high sensitivity and selectivity. As histamine occurred, Au-NPs could precisely and quantitatively be detected by the response of UV-vis and fluorescence spectrums with a limit of detection of 0.87 nM and 2.04 nM, respectively. Moreover, the significant colorimetric and fluorescence turn-on phenomenon of Au-NPs involved with histamine could be captured by eye vision. Both of the two detection methods of Au-NPs were successfully applied to the quantitative analysis of histamine in fresh salmon muscles, suggesting that the simplicity and rapidity of the Au-NPs dual-detection method may be applicable to the FDA to judge the freshness and spoilage of aquatic foods.

## Figures and Tables

**Table 1:** Analytical data for the colorimetric and fluorescence detection of histamine in real samples by Au-NPs probe, respectively.

Detection Methods		Spiked Concentration (μM)	Found Concentration (μM)	Recovery (%)	Precision (%)	Accuracy (%)
Au-NPs probe (this work)	Colorimetric detection	0.01	0.0102 ± 0.0010	102.13	9.32	2.13
		0.1	0.0997 ± 0.0049	99.67	4.95	−0.33
		1	1.0690 ± 0.0259	106.90	2.43	6.90
	Fluorescence detection	0.01	0.0096 ± 0.0004	96.01	4.46	−3.99
		0.1	0.1024 ± 0.0095	102.39	9.30	2.39
		1	1.0317 ± 0.0996	103.17	9.66	3.17

## Supplementary Materials

The following are available online at https://www.mdpi.com/2304-8158/9/3/316/s1, Scheme S1 Synthesis mechanism of Au-NPs, Figure S1 (A–D) TEM images of Au-NPs synthesized by different HAuCl_4_/Na_3_Ct precursor ratios (1:2, 1:4, 1:6, 1:8). (E) The average diameters and (F) UV-vis absorption spectra of Au-NPs. (G) Schematic illustration of the transformation process of Au-NPs size by citrate reduction.
