# Supplementary material for: Detection of Histamine Based on Gold Nanoparticles with Dual Sensor System of Colorimetric and Fluorescence
Source: Foods. 2020 Mar 9;9(3):316. doi: 10.3390/foods9030316 (PMC7143402; doi:10.3390/foods9030316)
Supplement: Supplementary file 1 [file foods-09-00316-s001.pdf]

Supporting Information

Detection of Histamine Based on Gold Nanoparticles with Dual Sensor System of Colorimetric and Fluorescence

Jingran Bi[12]*, Chuan Tian[12], Gong-Liang Zhang[12], Hongshun Hao[12], Hong-Man Hou[12]*

[1] School of Food Science and Technology, Dalian Polytechnic University, No. 1, Qinggongyuan, Ganjingzi District, Dalian, Liaoning 116034, People's Republic of China. E-mail: bijingran1225@foxmail.com.

[2] Liaoning Key Lab for Aquatic Processing Quality and Safety, No. 1, Qinggongyuan, Ganjingzi District, Dalian, Liaoning 116034, People's Republic of China. E-mail: bijingran1225@foxmail.com.

* Correspondence: bijingran1225@foxmail.com; Tel.: +86-411-8632-2020

Synthesis mechanism and preparation of Au-NPs

Au-NPs were successfully synthesized by reduced and capped via citrate in boiling water with an understandable phenomenon that the color of the reaction system changed from buff to wine red indicating colloidal gold with the intense surface plasmon resonance absorption at visible wavelengths was formed. The exact mechanism of Au-NPs formation was exhibited as Scheme 1: (a) [AuCl4]- employed citrate anion to coordinate an equilibrium by a fast substitution of a planar ligand Cl- with the citrate anion and formed an intermediate complex [AuCl3(C6H5O7)-2]-. (b) After the deprotonation of alcohol functional groups, the alcohol oxygen might rapidly coordinate with Au (III) and formed an axial pentacoordinate intermediate complex [AuCl3(C6H4O7)-2]-2. Moreover, by the rate-limiting decarboxylation and reduction, the complex was disintegrated into Au(I), decarboxylation molecules and other products(1). (c) Furthermore, subsequently, Au (0) atoms and Au(III) were concurrently formed via disproportionation of the multimolecular complex formed by Au(I) and decarboxylation molecules(2).

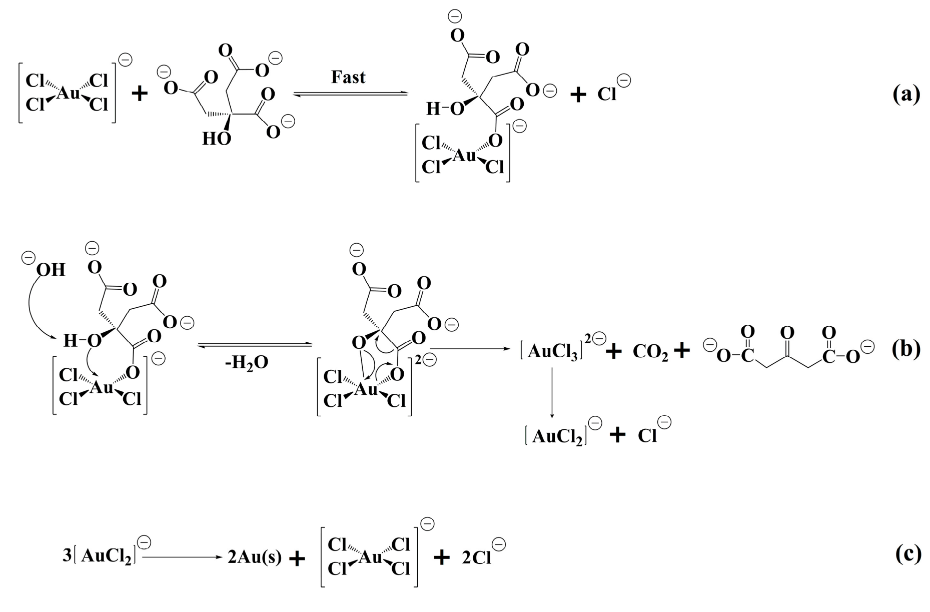

Scheme S1 Synthesis mechanism of Au-NPs

To explore the best sensor, different HAuCl4/Na3Ct precursor ratios (1:2, 1:4, 1:6, and 1:8) were applied in the synthetic system with 1.0 mM HAuCl4. As Fig.1 (A~D) shown, the morphology of Au-NPs transformed from oval to spherical with the apparent crystal lattice and surged nuclei number through the citrate reduction. The average diameters of Au-NPs were decreased from 17.2 to 10.7 nm that could be readily realized by the variation of Na3Ct addition (Fig. 1E). As the HAuCl4/Na3Ct precursor ratios get 1:6, the size of Au-NPs kept constant maintain invariable, suggesting the step of diffusion-controlled growth occurred following the exploding nucleation(3). Moreover, the optical evolution of the monomer structure during the reaction is a vital part to clarify the formation mechanism of NPs. Fortunately, by the morphology evolution obtained by TEM, the evolving regulars of UV-vis absorption spectra of Au-NPs production by different reaction solutions were also studied (Fig. 1F). By the low HAuCl4/Na3Ct precursor ratios (1:2), although the Au-NPs production possessed an undisturbed absorption peak at 520 nm, the value of peak height was relatively low with the substantial elongation, probably due to the morphology of Au-NPs tended to oval with a relatively large diameter similar to nanocrystals synthesis. Not unexpectedly, the prominent peak of 520 nm was gradually enhanced by the high precursor ratios, likely due to the end-product of Au-NPs was almost spherical dots in keeping with the TEM results. Additionally, all the Au ions being utilized and converted into Au-NPs, the absorbance of the 600~800 nm window gradually decreased with the high precursor ratios, implying the increase of monodispersity and the isotropic shape of Au-NPs. By the optical evolution, the fleeting nucleation process is obtained to illuminate the course of wire-shaped aggregates transform to spherical Au-NPs with high lattice energy. According to the classical Lamar model about the size distribution control of NPs(4), the formation process of Au-NPs is developed by nucleation-growth, initial rapid nucleation, and the diffusion-controlled growth process (Fig. 1G). There was no significant difference between the Au-NPs prepared from the precursor ratios of 1:6 and 1:8, both of which provided significant absorption at 520 nm. Considering that the sensing mechanism is related to the specific surface area and the intensity of plasmon resonance absorption, Au-NPs synthesized by the HAuCl4/Na3Ct precursor ratio of 1:6 with extremely small particle size, good monodispersity and strong UV-vis absorption at 520nm was chosen for follow-up determination of histamine.

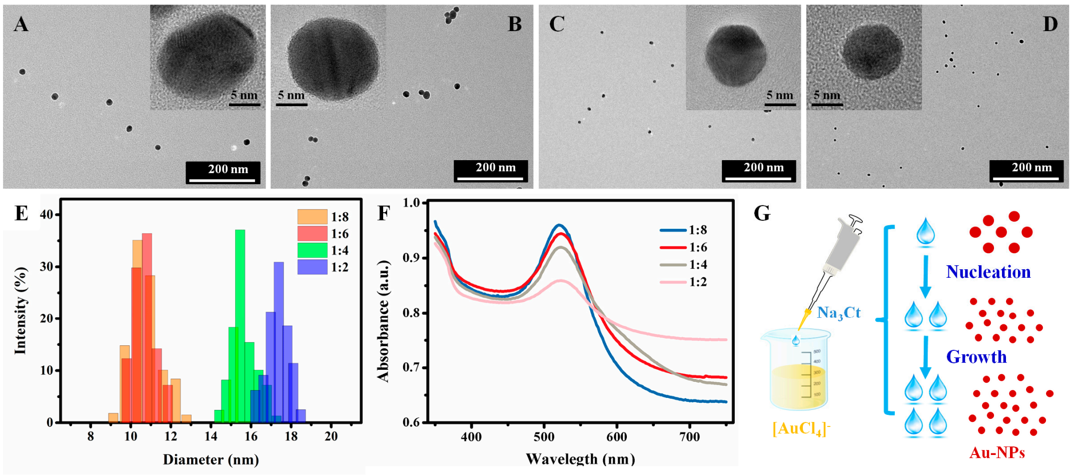

Figure S1 (A~D) TEM images of Au-NPs synthesized by different $HAuCl_4/Na_3Ct$ precursor ratios (1:2, 1:4, 1:6, 1:8). (E) The average diameters and (F) UV-vis absorption spectra of Au-NPs. (G) Schematic illustration of the transformation process of Au-NPs size by citrate reduction.

References

1. Soni, V, et al. (2007). Inorg Chim Acta, 360, 3141-8.
2. Ojea-JiméNez, Isaac, et al. (2010). J Phys Chem C, 114, 1800-4.
3. Piella, Jordi, et al. (2016). Chem Mater, 28, 1066-75.
4. Ji, X., et al. (2007). J Am Chem Soc, 129, 13939-48.
